# Conventional Wisdom versus Actual Outcomes: Challenges in the Conduct of an Ebola Vaccine Trial in Liberia during the International Public Health Emergency

**DOI:** 10.4269/ajtmh.16-1015

**Published:** 2017-05-01

**Authors:** Gregg S. Larson, Beth R. Baseler, Marie L. Hoover, Jerome F. Pierson, Jemee K. Tegli, Melvin P. Johnson, Mark W. S. Kieh, Laura A. McNay, Wissedi Sio Njoh

**Affiliations:** 1 Coordinating Centers for Biometric Research, Division of Biostatistics, School of Public Health, University of Minnesota, Minneapolis, Minnesota; 2 Leidos Biomedical Research, Inc., Frederick National Laboratory for Cancer Research, Frederick, Maryland; 3 Advanced BioMedical Laboratories, LLC, Cinnaminson, New Jersey; 4 National Institute of Allergy and Infectious Diseases, Rockville, Maryland; 5 Liberia-US Clinical Trials Partnership Program, Partnership for Research on Ebola Virus in Liberia (PREVAIL), Monrovia, Liberia

## Abstract

Clinical trials are challenging endeavors. Planning and implementing an investigational vaccine trial in Liberia, in the midst of an Ebola virus disease (EVD) epidemic that World Health Organization classified a public health emergency of international concern, presented extraordinary challenges. Normally, years of preparation and a litany of tasks lay the groundwork for a successful, randomized, blinded, placebo-controlled trial focused on safety and efficacy. Difficult research settings, unpredictable events, and other unique circumstances can add complexity. The setting in Liberia was especially problematic due to an infrastructure still badly damaged following a lengthy civil war and a very fragile health-care system that was further devastated by the EVD outbreak. The Partnership for Research on Vaccines in Liberia I EVD vaccine trial was planned and implemented in less than 3 months by a Liberian and U.S. research partnership, and its Phase II substudy was fully enrolled 3 months later. Contrasting conventional wisdom with trial outcomes offers an opportunity to compare early assumptions, barriers encountered, and adaptive strategies used, with end results. Understanding what was learned can inform future trial responses when disease outbreaks, especially in resource-poor locations with minimal infrastructure, pose a significant threat to public health.

## INTRODUCTION

By the time that World Health Organization (WHO) classified the Ebola virus disease (EVD) epidemic in West Africa a public health emergency of international concern on August 8, 2014, Liberia had declared its own state of emergency with 554 cases of confirmed, probable, and suspected EVD and 294 deaths.^1^^,^^2^ Building on its long-standing U.S. relationship, Liberia proposed a research partnership on EVD therapeutics and vaccines.^3^ The United States replied positively in early October, and shortly thereafter a WHO consultation endorsed a Phase III EVD vaccine trial.^4^ A formal agreement establishing an EVD research partnership was executed by the U.S. and Liberian governments on 19 November.^5^

### Phase II-III EVD vaccine trial.

**Figure 1.**
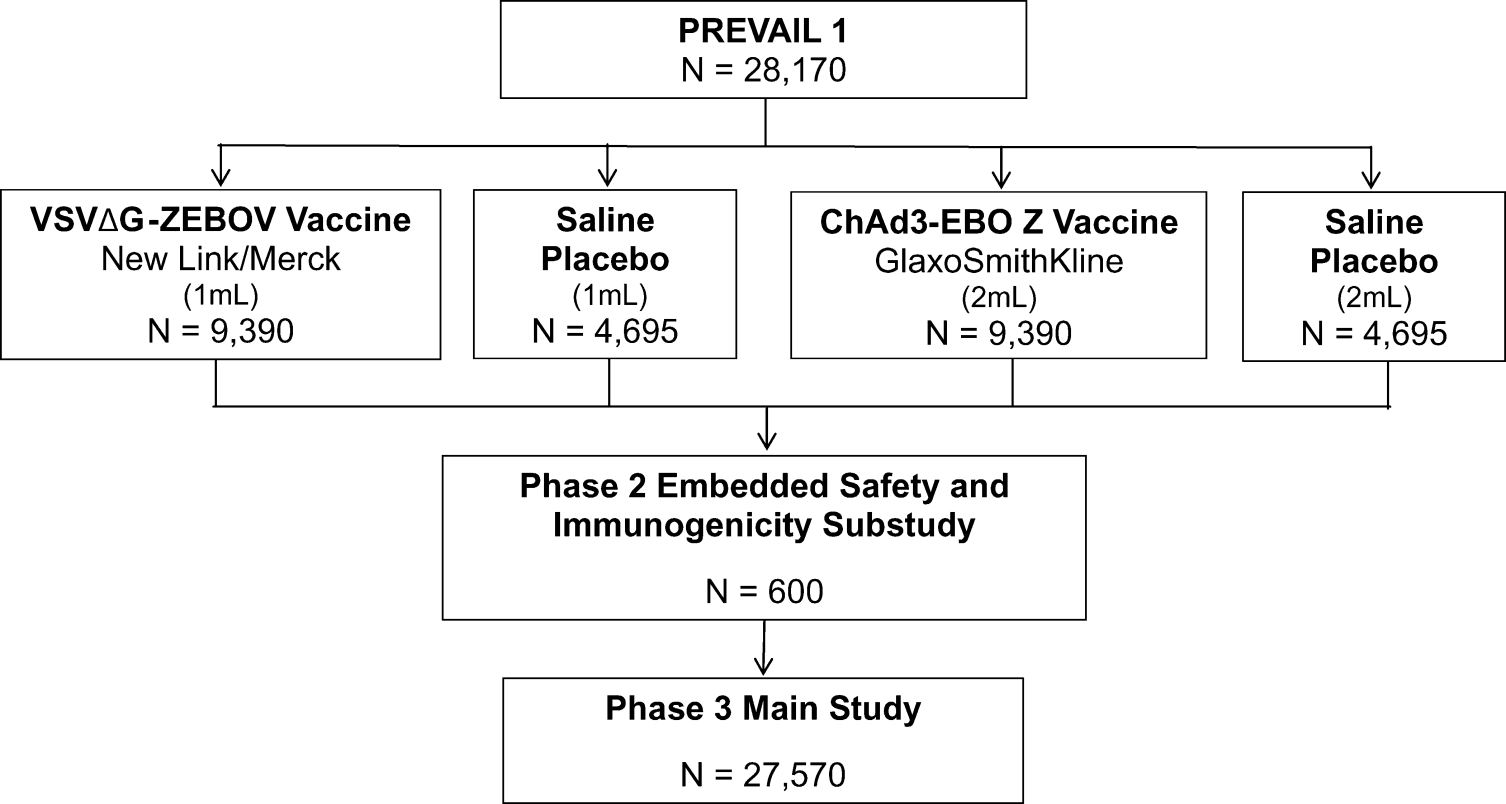
Clinical trial design for PREVAIL I.

The Partnership for Research on Vaccines in Liberia (PREVAIL I) was a randomized, double-blinded, placebo-controlled trial evaluating two investigational vaccines: the GlaxoSmithKline ChAd3-EBO Z vaccine and the New Link/Merck VSVΔG-ZEBOV vaccine (Figure 1

).^6^ Single-site accrual of 600 participants was planned for a Phase II substudy embedded within a Phase III main study. Based on Phase II results, Phase III could expand with 27,570 additional participants at up to 10 sites. Participants had to be at least 18 years of age, afebrile, neither pregnant nor breastfeeding, and without serious illness or past EVD history.

PREVAIL I would assess vaccine safety and efficacy prior to larger-scale vaccination efforts. The primary efficacy endpoint was EVD 21 days or more after randomization. The primary safety endpoint was serious adverse events within 30 days of vaccination. Blood would be drawn at vaccination, week 1, and month 1 visits for safety assays (aspartate aminotransferase, alanine transaminase, creatinine, potassium, D-dimer, partial thromboplastin time, complete blood count w/differential, platelet count, human immunodeficiency virus, and syphilis) that would reveal underlying disease or differences by arm. Week 1 and month 1 aliquots also would be frozen for later immunogenicity testing. Follow-up would end on a common closing date 12 months after enrollment.

Although earlier projections of EVD cases were dire,^7^^,^^8^ it was apparent by February 2015, when PREVAIL I opened, that declining incidence would render a Phase III study unachievable.^9^ Instead, the protocol was amended in mid-March to expand the safety substudy enrollment to 1,500 participants, add blood draws for immunogenicity testing at months 6 and 12, and postpone the Phase III study.^10^

### Conventional wisdom and actual outcomes.

Implementing a vaccine trial during an international health emergency was challenging. It required collaboration with WHO, national governments and regulators, the U.S. Embassy, clinical trialists, pharmaceutical representatives, community leaders, health-care institutions, and contractors. During the planning period, these entities brought a wide variety of educational and cultural backgrounds, clinical research and health-care experience, and other talents to the planning work. Because of its collaborative nature, the immediacy of the epidemic, and a resource-poor setting still recovering from a long civil war, the practitioners had to frequently reassess their approach.^11^ Unanimity was not always reached, and consensus building was relied on when speedy decisions were imperative. The Liberian and U.S. technical team began trial planning in early November, and the trial opened in less than 3 months, a remarkable achievement given the usual expectation of 1–3 years for randomized clinical trials.^12^^,^^13^

The Phase II safety substudy was successfully completed. However, conventional wisdom—usually a synthesis of preliminary assumptions, standard research practices, experience, and cultural perspectives—was not always borne out in implementation. With hindsight, these differences warrant examination and could be instructive in similar research endeavors. The following examples briefly characterize the conventional wisdom and actual outcomes for key aspects of the trial.

### Liberian approval of an EVD vaccine trial.

#### Conventional wisdom.

Given the unprecedented epidemic and high-level requests for U.S. assistance, Liberia would quickly embrace a U.S.-funded EVD vaccine trial.

#### Actual outcome.

Despite the growing toll of Liberian EVD cases (6,525) and deaths (2,697) by November 2014, Liberians did not immediately greet the trial with enthusiasm.^14^

Reluctant officials, lawmakers, academicians, and human rights activists expressed reservations associated with a) the infamous U.S. Tuskegee study and other African pharmaceutical trials, b) resistance to “experimentation” in a placebo-controlled trial, especially with Phase I vaccine results still pending,^6^ c) insistence that Liberia gain financially from vaccine commercialization, d) the lack of injury and death liability coverage, and e) uncertainty that all trial participants, and the larger population, could access an efficacious vaccine.^15^ Local media mirrored these reservations, fueling rumors and conspiracy theories.^16^ It became quickly evident that community engagement would be a condition precedent to successful trial implementation.^17^^,^^18^

Establishing the first vaccination clinic also was problematic. The preferred site, Monrovia’s large John F. Kennedy (JFK) Hospital, badly damaged during the war, had closed early in the EVD outbreak. Its governing board was unresponsive to appeals to designate space for renovation. Planning was redirected to Redemption Hospital, a small, free facility also closed early in the epidemic, but enthusiastic about reopening with the vaccination clinic. A local contractor renovated a small vaccination space at Redemption in 17 days.

Phase I EVD vaccine study results were eventually integrated in trial planning, and post-trial access to vaccines was assured.^10^ Interventions by Liberia’s Vice President, the U.S. Ambassador, and senior Liberian and National Institutes of Health (NIH) officials were necessary to finally obtain the approval of the Liberian President in late January.^13^

### Local regulatory oversight.

#### Conventional wisdom.

Like other west African countries, Liberia’s ethical and regulatory infrastructure lacked experience to review and oversee clinical research.^19^ Regulatory rules for the Liberia Medicines and Health Products Regulatory Authority (LMHRA) were not promulgated until February 2014.^20^ A National Research Ethics Board (NREB) was not established until December 2014.^21^

#### Actual outcome.

With the assistance of WHO, the U.S. National Cancer Institute (NCI) Institutional Research Board (IRB) and the U.S. Food and Drug Administration (FDA), necessary documents were submitted to the new NREB and LMHRA and received thorough reviews. The NREB augmented its membership, and both organizations prioritized their work and convened meetings to accommodate the dual pathways of Liberian and U.S. reviews. Reviews were completed in less than 2 weeks, and comments and follow-up requests reflected an understanding of research issues and responsibilities. Subsequently, the LMHRA conducted monitoring visits at both the pharmacy and clinic sites. Participant files, regulatory documentation, and written procedures were examined, complementing separate monitoring visits by a US NCI contractor.

The Liberian–U.S. ethical and regulatory collaboration strengthened mechanisms for future, timely clinical research oversight in Liberia, including other PREVAIL research.^22^^,^^23^ It also provided an opportunity to include Liberian and West African representation on the trial’s independent Data Safety and Monitoring Board (DSMB).

### Participant compensation.

#### Conventional wisdom.

International norms and ethical considerations would discourage participant compensation amounts deemed excessive or coercive.

#### Actual outcome.

After extended discussions of different compensation plans, Liberian research colleagues proposed a total of USD300 for nine participant visits during the 12-month follow-up period (Table 1

**Table 1:** Visit compensation, attendance, and laboratory collections for PREVAIL I (V1.0)*

			Visits attended		Laboratory collections†	
Visit type	Payment ($)	Visits expected‡	No.	%	No.	%
Vaccination	40	1,500	1,500	100.0	1,500	100.0
Week 1	20	1,500	1,487	99.1	1,487	100.0
Month 1	20	1,499	1,477	98.5	1,476	99.9
Month 2	10	1,496	1,460	97.6	NA	NA
Month 4	10	1,495	1,455	97.3	NA	NA
Month 6	30	1,494	1,459	97.7	1,457	99.9
Month 8	10	1,491	1,444	96.8	NA	NA
Month 10	10	1,489	1,435	96.4	NA	NA
Month 12	150	1,488	1,463	98.3	1,460	99.8
Total§	300	13,452	13,180	97.9	7,380	99.9

* Under V2.0 of the substudy, 24 of the 1,500 participants consented to additional visits and blood draws on Day 3, Day 10, and Week 2, but are not included in the table.

† Of visits attended.

‡ Visits expected were adjusted to reflect non-EVD deaths during follow-up (12) and reflect an additional 27 participants that missed their 12-month visits.

§ Totals for visits expected, visits attended, and laboratories collected include initial vaccination visit.

). This “inconvenience” compensation exceeded 60% of the UN gross domestic product per capita estimate for Liberia,^24^^,^^25^ and proportionately exceeded compensation offered Phase I EVD vaccine trial participants in other countries.^26^^–^^28^ Nevertheless, the Liberian and U.S. IRBs approved the longitudinal compensation proposal without comment. The approvals were consistent with shifting views regarding inducements in compensation models, especially the market model, for healthy research participants.^29^^,^^30^ Nearly USD450,000 in compensation was paid to PREVAIL I participants.

### Informed consent.

#### Conventional wisdom.

Pervasive illiteracy (adult literacy rate of 42.9%) and unwritten tribal languages (20% of Liberians speak English) would hinder efforts to obtain genuine informed consent.^31^^,^^32^

#### Actual outcome.

In a country dependent on a tradition of oral communication, an audiovisual presentation was first advocated, but later abandoned due to undependable power for audiovisual devices, technical troubleshooting challenges, and the need to re-film informed consent revisions. Instead, a low-tech approach was pursued; key informed consent concepts were identified, and NIH artists prepared 11 illustrative storyboards.^33^ Large-scale copies were hung in a 25-seat meeting room at Redemption. Liberian personnel summarized the storyboards in vernacular English, answered numerous questions, and arranged for translations if needed. Following the group discussion, each prospective participant met privately with a Liberian counselor to address any remaining concerns before making their decision. Enrolled participants received copies of their signed consent and the storyboards.

### Participant accrual and follow-up.

#### Conventional wisdom.

Redemption Hospital is located in New Kru Town, a poor neighborhood with a high incidence of EVD and health-care worker mortality, unemployment, drug use, and “fearbola.”^9^ Coupled with a waning epidemic (fewer than 10 cases/week by January 2015), reliance on traditional healers, and medical mistrust,^24^ sufficient accrual and follow-up would be difficult to achieve.

#### Actual outcome.

Recognizing the impediments to accrual, considerable time and effort were dedicated to social mobilization. Community, tribal, and religious leaders; government personnel; the media; and opinion leaders were encouraged to support the trial, public events were held, radio messages recorded, and a dedication celebration included Liberia’s Vice President.^34^

The trial opened on 2 February with more prospective enrollees at the health/febrile screening triage center than the daily vaccination capacity of 24. By mid-February, 348 future “reservations” had been issued for persons turned away. When accrual expanded to 1,500 in April, the enrollment period was lengthened, and the reservation system modified to accrue more women.^35^ Full enrollment was reached on April 30, 2015.

While there are no data on participant motivation, newspapers headlined compensation amounts.^24^ Anecdotal comments from participants and Redemption staff indicated that the visit payments had offset fears and attracted participants, especially young men. Despite later emphasis on recruiting women, 63% of the trial participants were men, and 68% of the men were less than 35 years old.

Among follow-up hurdles were the lack of residential addresses, varying living arrangements and family definitions, and rural ties leading to frequent absences. Participants received a photo ID card with 24-hour toll-free numbers to report side effects, such as fever or other adverse event symptoms, an appointment card for their next visit, and a compensation payment. A cadre of hired trackers met with participants monthly, documented events, and helped find those missing. Trackers used photos; baseline forms identifying landlords, neighborhood leaders, employers, and family; and their personal knowledge of the community. Open EVD treatment units were asked to report any vaccinated patients. Compensation amounts, coupled with the participant tracking, may also have significantly contributed to high visit attendance and low lost-follow-up rates, both of which were exceptional (Table1).

### Participant randomization and electronic data collection.

#### *Conventional wisdom*.

Electronic and mobile devices would be used, consistent with FDA guidance, for central randomization and remote data entry at clinic sites.^36^

#### *Actual outcome*.

Communication and power infrastructure shortcomings rendered central randomization, using telephone, PCs, or I-Pads, unfeasible. Instead, randomization occurred at vaccine administration. A filled syringe was drawn from two bags of 12 prepared daily, and a bar-coded label was torn off the syringe, scanned, and placed on the participant’s case report form to link the participant with the syringe contents. Each bag contained four pre-filled syringes for each of the three trial arms (four syringes with 1 mL of vaccine A and two matching syringes of 1 mL saline placebo, and four syringes with 2 mL of vaccine B and two matching syringes of 2 mL saline placebo—a 2:1:2:1 allocation). Staff might observe volume differences, but could not discern whether a syringe contained vaccine or its matching placebo.^6^

Participant source data were collected on paper using bar-coded informed consents and case report forms. The consents and forms were transferred daily to the old U.S. embassy compound, which had reliable power service and internet access, for transmittal to the Minnesota Statistical and Data Management Center using large, multifunction scanners. Upon confirmation of electronic capture in Minnesota, the paper originals were filed in secure cabinets. Quality assurance queries were transmitted back to Liberia. Site personnel corrected the original case report forms, and corrected forms were transmitted to Minnesota before refiling.

### Vaccine pharmacy.

#### *Conventional wisdom*.

Clinical site pharmacies would store and fill syringes with temperature-sensitive vaccines and placebo.^10^ With limited stability data, vaccinations were advised within 4 hours of filling.

#### *Actual outcome*.

Given EVD-related civil unrest in Monrovia,^37^ the war legacy, tensions between police and the general population, and the continued presence of UN peacekeepers, the U.S. government and vaccine manufacturers wanted a secure vaccine supply. A central pharmacy behind the high walls of the old U.S. embassy compound provided optimal security, limited access, reliable power for ≤ −60°C freezers, and necessary space within 1-hour driving time to Redemption Hospital and other potential Monrovia clinic sites.

The central pharmacy received and stored the two vaccines and saline placebo. Three assembly lines were equipped each morning with the two thawed vaccines, placebo, and matching rolls of bar-coded labels removed from a safe. Syringes were aseptically filled by U.S. or Liberian pharmacy staff under biocontainment hoods. Four filled and labeled syringes were collected from each line. The 12 syringes were scanned to confirm the correct randomization mix and put in a randomization bag. Two bags of 12 were placed in a temperature-controlled transport cooler and delivered to Redemption via armored embassy vehicles.

The result was a nearly flawless pharmacy operation with only a single known instance of labeling error. The error was recognized and corrected before the syringes left the pharmacy.

### Safety laboratory.

#### *Conventional wisdom*.

Central safety laboratories would receive and analyze specimens from multiple vaccination clinics, minimizing equipment, space, and staffing needs.

#### *Actual outcome*.

Transportation turnaround time, unobtainable dry ice, and a prohibition on possibly infectious specimens on the U.S. embassy grounds limited central location choices. Despite many challenges, Redemption became a default clinic safety laboratory. Space was limited, new generators were needed for primary and backup power, battery backup of instruments was required, and line conditioners were necessary to smooth power fluctuations.

In addition, instrument selection was restricted to tabletop models able to read bar codes on pre-labeled collection tubes and kits, perform assays as closed systems to preclude release of infectious material,^6^ operate at 220 voltage, and transmit electronic data. Short lead times were essential for procurement. Other challenges included an unreliable wireless network that supported a custom information system connecting instruments, durable laptops, and backup hard drives; varying levels of staff familiarity with the instruments; and vendor refusal to travel to Liberia to troubleshoot problems.

The laboratory had an excellent specimen collection rate (Table 1) and, on peak days, processed over 100 specimens. Specimens collected for immunogenicity testing were aliquoted, frozen, and transported weekly in coolers to a remote laboratory for longer-term storage and analysis.

### Liability insurance coverage.

#### Conventional wisdom.

Commercial liability insurance would be purchased to address Liberian concerns about injury or death claims attributable to trial participation.

#### Actual outcome.

Sponsor treatment of clinical trial injuries may be offered,^38^ but liability compensation for injury or death is less common. Responding to requests for such coverage, a quote was obtained from a commercial carrier already providing EVD research coverage in Sierra Leone. Commercial insurance offered competitive costs, spread risk over a large pool, and could be quickly activated. The estimated cost of USD40,000–300,000 depended on the coverage amount and deductibles.

However, Liberians lacked confidence that an international carrier would provide timely, independent claim review and compensation. They preferred a broad scope, self-insurance fund, capitalized by the U.S. government and pharmaceutical firms, and administered by Liberia’s National Social Security and Welfare Corporation. Extended review ensued, but differences regarding the source and amount of capitalization, coverage of disease unrelated to the trial, claim adjudication, and dispute arbitration proved insurmountable.

The trial was underway when insurance discussions began, and follow-up concluded in May 2016 without agreement, but also without any claims of injury or death. Anticipating similar liability demands in future disease outbreaks, the World Bank has proposed a pandemic emergency facility that could frontload insurance funds.^39^

### Supply, shipping, and staffing logistics.

#### Conventional wisdom.

U.S. government and contractor support would ensure timely fulfillment of trial supply and shipping needs, but Liberian health-care personnel would be reluctant to resume work during the epidemic at facilities that had closed due to previous EVD transmission.

#### Actual outcome.

Procurement required careful screening of requests, identification of vendors, and confirmation of inventories and lead times. Round-the-clock supply demands for the pharmacy center, vaccination clinic, and safety laboratory soon overwhelmed the NIH contractor. Additional personnel were assigned and standard documentation was developed to track requests, generate pallet lists and commercial invoices, and manage inventory. An early option, military airlift, proved unfeasible because of military priority issues. Instead, supplies were consolidated at a central location in Maryland, and semiprivate charters began air shipments on December 24, 2014; they continued 3 times/week until May 2015 when frequency fell to 1–2/week and shipments were shifted to commercial carriers. U.S. embassy personnel and vehicles met vaccine and supply shipments, resolved airport clearance issues, and transported contents to local destinations.

Another subcontractor with a U.S. and Liberian presence, familiar with federal acquisition regulations and Liberian laws, assisted with staff hiring and payroll management. Liberia’s weak health-care system was exacerbated by the epidemic; 8% of health-care workers died, including five of Liberia’s 51 doctors and 78 of 978 nurses.^40^ Most of the health facilities had closed by the fall of 2014. When Redemption Hospital reopened in late January, 2015, Liberian health-care personnel were ready to return out of economic necessity, EVD incidence had significantly declined, and triage and infection control measures were introduced at the vaccine clinic. PREVAIL hired 85 staff (including six doctors and 13 nurses) and 319 part-time, social mobilization staff. Their performance exceeded expectations, was recognized by the DSMB, and was emblematic of future PREVAIL research capabilities in Liberia.

## DISCUSSION

Viral hemorrhagic fever outbreaks have been widely reported in recent years. Thirty-four EVD outbreaks have occurred since 1976, a record suggesting that the 2014 occurrence will not be the last.^41^ Circumstances in Liberia, including the epidemic’s scale and delayed global response, the early research status of investigational vaccines, missing infrastructure, cultural differences, and the complexities of a multi-party undertaking, may not all be encountered elsewhere. However, future clinical trial endeavors elsewhere could benefit from experience gained in this trial.

Conventional wisdom was often incorrect or not applicable, and outcomes differed from expectations. Lessons learned during the PREVAIL I trial included the following:

The full support of the U.S. government and the U.S. Embassy was indispensable and extraordinary in responding to staffing, transportation, logistics, and participant compensation needs.
Despite Liberian pleas for rapid assistance, building the corresponding political and institutional will, in the presence of media fear mongering and public suspicion, required a broad community engagement effort.
Advancing other Phase I and II vaccine research would speed implementation of Phase III trials during disease outbreaks.
Considerable strengthening of local ethical and regulatory agencies was required for them to undertake meaningful oversight of clinical research.
Generous compensation may be necessary to enroll otherwise healthy, but reluctant, participants in clinical trials.
Given widespread illiteracy and a tradition of oral communication, the simplest solutions to disseminating or collecting information were often the best and most efficient.
Creative flexibility was essential to overcome electric power, communication, and transportation obstacles that precluded some standard clinical trial practices.
A lack of confidence in governmental authorities to maintain public order during an epidemic may necessitate security measures that impact trial implementation.
International agreement on a financial mechanism to manage clinical trial liability coverage would be reassuring to governments and trial participants.
Unemployed health-care personnel wanted to return to work as EVD incidence subsided and the likelihood of transmission diminished.
Projections of disease incidence and an epidemic’s trajectory are inexact and can significantly affect research plans.

The PREVAIL I experience furthered subsequent PREVAIL EVD studies in Liberia and West Africa, including treatment, natural history, and viral persistence investigations that will continue to inform future research. These investigations have led to substantial investment in clinical facilities at JFK Hospital, Redemption Hospital, and local care clinics in Liberia; research staff training in Liberia, Guinea, and Sierra Leone; research ethics and regulatory agencies in Liberia; and expanded EVD laboratory capabilities at Redemption and the Liberian Institute for Biomedical Research.
